# The Effect of COVID‐19 on Incident Diabetes in Pediatric Patients: Findings From the National COVID‐19 Cohort Collaborative (N3C)

**DOI:** 10.1155/pedi/3545727

**Published:** 2025-12-05

**Authors:** Rachel Wong, Talia Wiggen, Margaret A. Hall, Steven G. Johnson, Jared D. Huling, Lindsey E. Turner, Kenneth J. Wilkins, Hsin-Chieh Yeh, Til Stürmer, Carolyn T. Bramante, Zachary Butzin-Dozier, John B. Buse, Jane Reusch, N3C Consortium

**Affiliations:** ^1^ Department of Biomedical Informatics, Stony Brook University, Stony Brook, New York, USA, stonybrook.edu; ^2^ Department of Internal Medicine, Stony Brook University, Stony Brook, New York, USA, stonybrook.edu; ^3^ Clinical and Translational Science Institute, University of Minnesota, Minneapolis, Minnesota, USA, umn.edu; ^4^ Department of Hematology and Medical Oncology, Emory University, Atlanta, Georgia, USA, emory.edu; ^5^ Institute for Health Informatics, University of Minnesota, Minneapolis, Minnesota, USA, umn.edu; ^6^ Division of Biostatistics and Health Data Science, School of Public Health, University of Minnesota, Minneapolis, Minnesota, USA, umn.edu; ^7^ Biostatistics Program, Office of Clinical Research Support, Office of the Director, National Institute of Diabetes and Digestive and Kidney Diseases, National Institutes of Health, Bethesda, Maryland, USA, nih.gov; ^8^ Department of Medicine, Johns Hopkins University, Baltimore, Maryland, USA, jhu.edu; ^9^ Department of Epidemiology, Johns Hopkins University, Baltimore, Maryland, USA, jhu.edu; ^10^ Department of Epidemiology, Gillings School of Global Public Health, University of North Carolina at Chapel Hill, Chapel Hill, North Carolina, USA, unc.edu; ^11^ Division of General Internal Medicine, University of Minnesota Medical School, Minneapolis, Minnesota, USA, umn.edu; ^12^ Division of Biostatistics, University of California, Berkeley, Berkeley, California, USA, berkeley.edu; ^13^ Division of Endocrinology and Metabolism, Department of Medicine, University of North Carolina School of Medicine, Chapel Hill, North Carolina, USA, unc.edu; ^14^ Division of Endocrinology, Metabolism and Diabetes, University of Colorado Anschutz Medical Campus, Aurora, Colorado, USA, ucdenver.edu; ^15^ National COVID Cohort Collaborative (N3C), Bethesda, Maryland, USA

## Abstract

**Objective:**

Studies showing increased diabetes incidence in pediatric patients after COVID‐19 are from data early in the pandemic, and some studies found conflicting results. Our objective was to evaluate trends in pediatric diabetes incidence and whether COVID‐19 was associated with increased risk across viral variant periods.

**Research Design and Methods:**

We conducted a retrospective cohort study using National COVID‐19 Cohort Collaborative data to evaluate incident diabetes risk among COVID‐19‐positive pediatric patients compared to COVID‐19‐negative patients or controls with acute respiratory illness. Cohorts were weighted on demographics, data site, and body mass index percentile. The primary outcome was the cumulative incidence ratio (CIR) of incident diabetes for each viral variant era.

**Results:**

There was no difference in the risk of incident diabetes in pediatric patients after COVID‐19 compared to patients in COVID‐19 negative or ARI control groups during any of the viral variant periods (e.g., ancestral period CIR 1.03, 95% CI 0.65–1.41). The predominant subtype of incident diabetes was T2D. Incidence rates over time followed a U‐shaped curve, with the highest incidence in the ancestral variant period.

**Conclusions:**

COVID‐19 was not associated with an increased risk of diabetes in pediatric patients. Incidence rates were highest early in the pandemic, and mirrored patterns of pandemic‐era healthcare utilization. The predominance of incident T2D subtype is concerning for the adverse effects of pandemic‐related lifestyle changes among pediatric patients.

## 1. Introduction

The COVID‐19 pandemic has had long‐term health consequences in the pediatric US population, with patients suffering from the postacute sequelae of infection [1–3], detrimental changes in health behaviors [4–6], and increased risk of chronic diseases such as diabetes [7]. There have been multiple studies done in large cohorts that show an increased risk of type 1 diabetes (T1D) after COVID‐19 compared to pediatric patients without COVID‐19 or with non‐COVID‐19 respiratory infection. Proposed pathophysiologic mechanisms for the increase in diabetes risk include direct damage to the beta cells due to SARS‐CoV‐2, pancreatic injury due to inflammation, and development of autoimmunity [8–11]. Studies conducted early in the pandemic have also shown increases in the incidence of pediatric type 2 diabetes (T2D) compared to prepandemic rates [12–15], and increased risk after COVID‐19 compared to other non‐COVID‐19 respiratory infections [16]. Increased risk may be attributable to factors such as increased processed food consumption, psychosocial stress, and decreases in physical activity [6], which disproportionately affected low‐income and Black and Latino households [4].

The risk of incident T1D after COVID‐19 in pediatric patients has been studied in multiple cohorts. In a study using data from 27 million individuals in the Cerner Real World Data, 22.8% of which were pediatric, T1D risk was elevated after COVID‐19 compared to patients without COVID‐19 (odds ratio [OR] 1.42, 95% CI 1.38–1.46); the risk was highest in the youngest patients (age 0–1), but it was elevated across all stratified age groups [17]. A study using Bavarian claims data from 1.8 million children reported an age‐ and sex‐ adjusted hazard ratio (HR) for the development of T1D of 1.57 (95% CI 1.32–1.88) with COVID‐19 compared to historical controls [18]. Given the potential role of viral and bacterial infection in the development of T1D in pediatric patients [19, 20], studies have also compared incident T1D risk after COVID‐19 to patients with non‐COVID‐19 respiratory infections. Data from the TriNetX database showed that the risk of T1D in propensity score‐matched patients with COVID‐19 vs. non‐COVID‐19 respiratory infection was elevated at one (HR 1.96, 95% CI 1.26–3.06), three (HR 2.10, 95% CI 1.48–3.00), and 6 months (HR 1.83, 95% CI 1.36–2.44) [21]. However, some studies have not shown any difference in T1D incidence between populations with and without COVID‐19, including another study using the TriNetX data (OR 1.15, 95% CI 0.68–1.65) [22]. In a cohort analysis using the national registry in Scotland, SARS‐CoV‐2 infection >30 days prior was not associated with T1D incidence (risk ratio [RR] 0.86, 95% CI 0.62–1.21), and the data did not support a causal effect of SARS‐CoV‐2 [23].

While most studies have focused on incident T1D, there have also been studies reporting increased risk of T2D and all diabetes among pediatric patients. A recent study using TriNetX data also reported increased incident T2D risk at one (RR 1.55, 95% CI 1.28–1.89), three (RR 1.48, 95% CI 1.24–1.76), and six (RR 1.58, 95% CI 1.35–1.85) months [16]. A meta‐analysis of 9 studies showed that in patients with age <18, the relative risk of all diabetes was 1.72 (95% CI 1.19–2.49) compared to non‐COVID‐19 patients [24]. The CDC studied the risk of new diabetes diagnoses of all types, and reported that in age‐ and sex‐matched patients, risk was higher after COVID‐19 in two databases (IQVIA: HR 2.66, 95% CI 1.98–3.56; HealthVerity: HR = 1.31, 95% CI 1.20–1.44) compared to non‐COVID‐19 patients and in one database (IQVIA, HR 2.16, 95% CI 1.64–2.86) compared to prepandemic non‐COVID‐19 acute respiratory infection (ARI) [25].

The issue of whether there is an increase in incident diabetes after COVID‐19 is still unclear in pediatric patients, but it is important due to the significant morbidity and need for health care associated with diabetes diagnosis [26]. In contrast to the adult population, where COVID‐19 has been associated with increased diabetes incidence [27, 28], children and adolescents experience lower severity of symptoms and better outcomes in acute COVID‐19 than adults [29, 30]. The data on most studies of incident diabetes in pediatric patients are from early in the pandemic, and also do not reflect the evolution in treatment and prevention strategies that became available throughout the pandemic [31–33]. Recent vigilance around COVID‐19 transmission has diminished, and it is important to understand whether the incidence and risk differ by viral variant, and to define the contemporary risk with newer viral variants.

We conducted a retrospective cohort study using National COVID‐19 Cohort Collaborative (N3C) data to evaluate the risk of incident diabetes among pediatric patients with COVID‐19 compared to patients with negative COVID‐19 testing or acute respiratory illness across viral variant time periods.

## 2. Materials and Methods

### 2.1. Study Design and Cohort

The National COVID‐19 Cohort Collaborative (N3C) data includes electronic health record data from 22.1 million patients from 83 sites around the US [34–36]. We included pediatric patients <18 years with ≥2 visits in the 2 years prior to the index date who had no prior evidence of diabetes, as defined by absence of a preindex diabetes diagnosis code, HbA1c ≥ 6.5 mg/dL, or glucose ≥ 200 mg/dL. We used two control groups: (1) non‐COVID‐19 patients with a negative COVID‐19 test and no evidence of ARI, and (2) patients with ARI. ARI was defined as patients without COVID‐19 who had a positive influenza test or diagnosis code for either bacterial or viral bronchitis or pneumonia, acute upper respiratory infection (AURI), or influenza [27]. The index date was the date of the first COVID‐19 diagnosis by laboratory test or diagnosis code for positive patients, or the date of the first negative SARS‐CoV‐2 test or ARI diagnosis for the two control groups. We conducted analyses for four viral variant eras based on dates: Ancestral 3/1/2020‐9/30/2020, Alpha 10/1/2020‐6/30/2021, Delta 7/1/2021‐11/30/2021, and Omicron 12/1/2021‐4/1/2023 [37]. Individuals were included as controls and were censored if a new index event occurred in any viral variant period; they could however be included in another cohort if they had a new index event (e.g. COVID‐19 negative controls who developed ARI or COVID‐19 were censored from the COVID‐19 negative group for that variant era, but could be included in the ARI or COVID‐19 group for a different variant era). To ensure data quality, we excluded data partner sites that did not submit data between June 6, 2022 and December 15, 2022, sites where ≥90% of patients were missing data on birthdate, sites missing >25% of serum creatinine or white blood cell lab results for COVID‐19 hospitalizations, or sites with >10% of COVID‐19 hospitalizations reporting a length of stay >200 days. Two sites were excluded that had an unusual age distribution of COVID‐19‐negative tests. We also excluded patients who had visit days greater than 2 standard deviations above the mean (141 days) to exclude patients in long‐term care.

### 2.2. Outcomes and Covariates

The primary outcome of the study was incident diabetes, as defined by HbA1c > 6.4, or diagnosis code after the index date. Diabetes was defined by concept sets of codes for T1D, T2D, unspecified or diabetes due to other causes developed by subject matter experts in the N3C Diabetes and Obesity Domain Team [38–40]. For patients with records for both T1D and T2D, diabetes subtype was adjudicated based on the presence of a diagnosis code of diabetic ketoacidosis (DKA) to indicate T1D, or ≥50% of the diagnosis for a subtype in the year following the index date [41]. Hospitalization was defined as an inpatient visit 15 days prior to or 15 days after the index date, and systemic corticosteroid use was defined by a record of a systemic corticosteroid medication within 30 days of the index date.

### 2.3. Statistical Analysis

Study populations were balanced using stabilized inverse probability of treatment weighting, estimated with a multinomial logistic regression on data partner, age, gender, race/ethnicity, BMI percentile [42], and the interaction of data partner with these demographic features using a logistic regression model in SparkR. We assessed the balance between study and control populations using the standardized mean difference, and all analyses incorporated the inverse probability of treatment weights. For the primary analysis, we estimated the cumulative incidence function of incident diabetes for COVID‐19 positive, ARI, and COVID‐19 negative patients in each variant time period. We accounted for death as a competing risk with the Aalen–Johansen estimator and prodlim competing risk model [43, 44]. We calculated the cumulative incidence ratio (CIR) of incident diabetes at 180, 365, 548, and 730 days from the index date [27]. Individuals were censored for death, end of the study period, or for loss to follow‐up, defined as the last recorded observation, laboratory value, or visit in the data.

Data access and analysis were conducted using Python 3.6 and Python 3.9 and R 3.5.1 and data release version 112 (2023‐02‐23) using Palantir’s Foundry platform (2021, Denver, CO), a secure analytics enclave housing the N3C data. The Stony Brook University Office of Research Compliance determined that the study did not constitute human subjects research.

## 3. Results and Discussions

**Figure 1:**
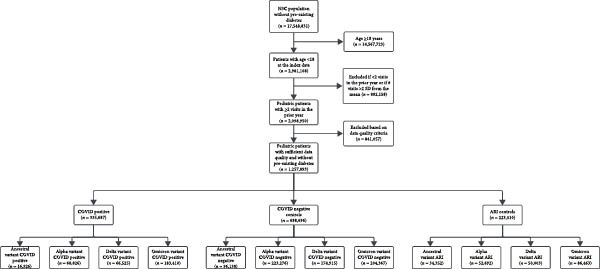
Flow diagram of study cohorts.

Of the 2,981,108 pediatric patients in N3C without pre‐existing diabetes, 1,257,893 (42.2%) were included in the final cohort. The final number of pediatric patients in the COVID‐19 positive, and COVID‐19 negative, and ARI control groups that were included in the analysis are shown for each viral variant (Figure 1). Tables showing the unweighted and weighted characteristics of the cohorts for each viral variant era are included in Tables S1–S4. Overall, the groups were balanced with respect to age categories, gender, race/ethnicity, and BMI percentile after weighting, with standardized mean differences less than 0.1. There was a higher rate of hospitalization in the COVID‐19 negative patients (12.5%, 8.4%, 7.1%, and 8.9% for the ancestral, Alpha, Delta and Omicron variant eras respectively) than the COVID‐19 positive (4.2%, 2.6%, 2.7% and 2.9%) and ARI patients (3.0%, 1.8%, 2.1% and 2.8%). Corticosteroid use was also higher for COVID‐19 negative patients (17.2%, 12.3%, 9.4% and 10.7%) than COVID‐19 positive (3.7%, 3.1%, 4.1% and 5.2%) and ARI patients (7.1%, 6.5%, 8.0% and 9.0%). Corticosteroid use was higher in ARI patients than in COVID‐19 positive patients, but rates of hospitalization were closer between the two groups.

For each viral variant era, the rates of incident diabetes outcomes per 1000 person‐years (PY), and for T1D and T2D subtypes, are shown in Table 1. We found the highest rates of incident diabetes diagnoses during the ancestral variant era overall (1.76/1000 PY) and for COVID‐19 positive (1.68/1000 PY) and COVID‐19 negative (1.86/1000 PY), and ARI (1.59/1000 PY) control groups. Approximately half of the diagnoses of incident diabetes were of the T2D subtype for all variant periods, with the highest percentage (57%) in the ancestral variant era. Pediatric patients with incident T2D were predominantly from older age groups, higher BMI, and Black or Hispanic race/ethnicity. Results could not be reported for T1D and T2D subtypes due to privacy requirements under the data use agreement for results with small patient numbers, but can be accessed in the N3C Data Enclave. Of the 96 patients with both T1D and T2D diagnosis codes, 12 patients were not included in subtype incidence rates in Table 1 because they had equal numbers of T1D and T2D diagnoses and no diagnosis of DKA.

**Table 1:** Weighted incidence rate of diabetes by viral variant for COVID‐19 positive and COVID‐19 negative and ARI controls in 1000 person‐years.

Variant and diabetes type	Overall	COVID‐19 positive	COVID‐19 negative	ARI
Ancestral variant				
Diabetes^a^	1.76 (1.73–1.79)	1.68 (1.18–2.18)	1.86 (1.61–2.11)	1.59 (1.01–2.16)
Type 1 diabetes	0.35 (0.35–0.35)	0.17 (0.00–0.34)	0.38 (0.27–0.49)	0.38 (0.17–0.59)
Type 2 diabetes	1.01 (0.99–1.03)	1.05 (0.68–1.42)	1.02 (0.84–1.21)	0.96 (0.46–1.45)
Alpha variant				
Diabetes^a^	1.52 (1.51–1.53)	1.29 (1.06–1.51)	1.67 (1.49–1.84)	1.36 (0.99–1.73)
Type 1 diabetes	0.39 (0.39–0.39)	0.30 (0.19–0.42)	0.40 (0.32–0.49)	0.51 (0.31–0.71)
Type 2 diabetes	0.80 (0.80–0.80)	0.74 (0.57–0.91)	0.89 (0.76–1.01)	0.61 (0.34–0.89)
Delta variant				
Diabetes^a^	1.23 (1.22–1.24)	1.23 (0.92–1.54)	1.38 (1.15–1.62)	0.83 (0.54–1.12)
Type 1 diabetes	0.35 (0.35–0.35)	0.48 (0.29–0.67)	0.28 (0.18–0.38)	0.34 (0.15–0.53)
Type 2 diabetes	0.59 (0.58–0.60)	0.41 (0.25–0.57)	0.79 (0.61–0.96)	0.34 (0.16–0.52)
Omicron variant				
Diabetes^a^	1.44 (1.42–1.46)	1.32 (1.08–1.55)	1.64 (1.31–1.98)	1.37 (0.90–1.85)
Type 1 diabetes	0.38 (0.38–0.38)	0.36 (0.23–0.48)	0.32 (0.19–0.46)	0.56 (0.27–0.85)
Type 2 diabetes	0.78 (0.77–0.79)	0.72 (0.55–0.90)	0.88 (0.63–1.14)	0.75 (0.38–1.11)

^a^Diabetes includes all types (T1, T2D, unspecified diabetes, and diabetes due to other causes).

**Figure 2:**
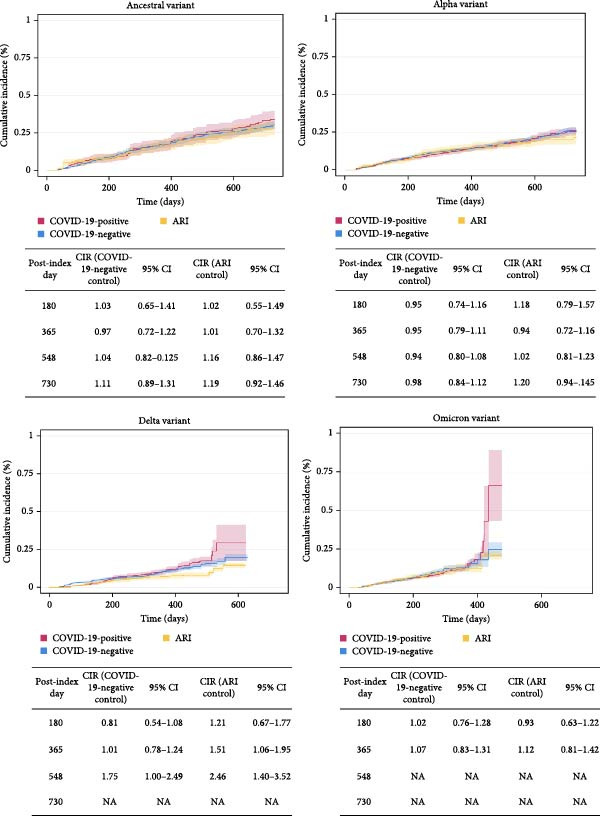
Weighted cumulative incidence of diabetes in pediatric patients after COVID‐19 compared to COVID‐19 negative and ARI controls by COVID‐19 variant and time period.

The weighted cumulative incidence curves and CIR at half‐year intervals for incident diabetes for pediatric COVID‐19 positive, COVID‐19 negative, and ARI patients are shown in Figure 2. There was no difference in risk of incident diabetes in patients with COVID‐19 compared to COVID‐19 negative or ARI controls for the ancestral, Alpha, and Omicron viral variant time periods. In the Delta variant era, there was no difference at 180 days, but a statistically significant difference in risk at 365 and 548 days compared to the ARI group and at 548 days compared to the COVID‐19 negative group. A potential mechanism for this slightly higher risk could be adipocyte hypertrophy from lifestyle changes during lockdowns in 2020–2021. Hypertrophic adipocytes are more inflammatory and are at greater risk for fibrosis and dysfunction, and thus may have been more primed to cause insulin resistance by the time the Delta variant was predominant [45]. However, these results should be interpreted with caution, as risk may be overestimated due to unequal and potentially informative censoring of patients in the COVID‐19 positive group; patients in the COVID‐19 negative group were censored if they developed ARI or COVID‐19, and patients in the ARI group were censored if they developed COVID‐19. Selection or survivor bias may be present, where patients with greater comorbidity burden and/or greater frequency of healthcare utilization may be disproportionately censored from the COVID‐19 negative and ARI groups, which may exclude potential incident diabetes cases and shift estimates away from the null hypothesis [46, 47]. Furthermore, given the increased risk of T2D with older age in pediatric individuals, the unequal censoring between groups could increase the hazard in the COVID‐19 group. Additionally, the proportion of COVID‐19 censored for loss to follow‐up was higher than the COVID‐19 negative or ARI groups, which could also affect the risk estimates at longer follow‐up periods. The number at risk by viral variant for COVID‐19 positive, COVID‐19 negative and ARI patients are shown in Tables S5 and S6, and the weighted counts of censoring events by viral variant and group are shown in Table S7. The uncensored proportion of patients in each cohort with incident diabetes is shown in Table S8. The cumulative incidence curves for death, incident diabetes by hospitalization status, and cumulative incidence by variant at 180, 365, 548, and 730 days, and numbers at risk can be found in Figures S9, S10, and Table S11.

**Figure 3:**
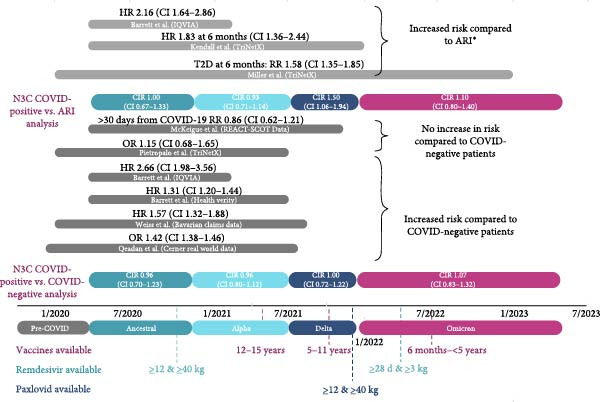
Results of studies comparing risk of incident diabetes in pediatric patients in COVID‐19 positive vs. COVID‐19 negative vs. ARI control groups. HR denotes hazard ratio; RR, rate ratio; OR, odds ratio; CIR, cumulative incidence risk ratio at 1 year after index date. Pietropalo et al. reported ORs as the risk of incident diabetes in COVID‐19‐negative patients compared to COVID‐19‐positive patients.  ^∗^ARI is used to represent non‐COVID‐19 respiratory infections.

The timeline of studies comparing the risk of incident diabetes in pediatric COVID‐19 positive vs. ARI and non‐COVID‐19 controls shown in Figure 3 shows the time period over which the data was analyzed by each study, with respect to the different variant eras and the current N3C analysis. The reported primary outcome of incident diabetes risk is shown for each study as either an HR, OR, or relative RR. The CIR of incident diabetes at 1‐year in the N3C analysis is shown for each viral variant era. The dates of availability for remdesivir, Pfizer‐BioNTech COVID‐19 vaccination, and nirmatrelvir‐ritonavir are indicated based on the Emergency Use Authorization (EUA) issuance by the US Food and Drug Administration [48].

There has been significant concern regarding an increased risk of diabetes in pediatric patients associated with the COVID‐19 pandemic, both related to potential pathophysiologic mechanisms related to infection and pandemic‐related lifestyle changes. Most studies were conducted using data from early in the pandemic during the ancestral and Alpha variant eras; our data also show the highest rates of overall incident diabetes diagnoses during the ancestral period. The incidence rates of T1D remain relatively stable over all variant periods, suggesting that the fluctuation in incidence is primarily driven by T2D diagnosis. Interestingly, the overall rates of T2D follow a pattern of the highest incidence in the ancestral period (1.01/1000 PY), then decreasing incidence in the Alpha (0.80/1000 PY) and Delta (0.59/1000 PY), and an increase in the Omicron period (0.78/1000 PY). These findings suggest a surge of new diagnoses early in the pandemic, a time during which routine healthcare utilization decreased [49, 50] and individuals interacting with the health system were likely more chronically or acutely ill, or presenting for COVID‐19‐related care. The early pandemic time frame also coincides with concerning reports of rising pediatric T2D incidence, especially among Medicaid enrollees [15] and males, who may have increased cardiometabolic risk at lower thresholds of visceral adiposity [14, 15, 51]. However, this early rise in incidence is not consistent across viral variant periods in our data, which suggests that while COVID‐19 or pandemic‐related changes may have resulted in earlier diagnoses of those at risk, there is a subsequent dip and leveling off of incidence rates. Reddy et al. [52] also found that after a sharp increase in diabetes diagnoses after COVID‐19, there was a period with decreased incidence, and that the initial spike in diabetes diagnoses may be related to increased interaction of patients with COVID‐19‐related or more severe non‐COVID‐19 illness with the healthcare system. Other studies on pandemic‐era healthcare utilization demonstrate a U‐shaped curve in the utilization of routine health services [53, 54]; the pattern of T2D incidence in our data mirrors this pattern, and suggests that the incidence rate of T2D in the Omicron period may be more reflective of the postpandemic baseline rate. Alternatively, the increased incidence during the Omicron period could represent the late effects of pandemic‐related lifestyle changes, as T2D can take time to develop. Our findings highlight the importance of longitudinal follow‐up of epidemiologic trends in T2D incidence in the pediatric population.

Among pediatric patients, diabetes prevalence has been predominantly of the T1D subtype [55]. The estimated prevalence of T1D in 2017 was 2.15/1000 youths, with an absolute of 0.67/1000 youths between 2001 and 2017; for T2D, the prevalence was 0.67/1000 youths with an absolute increase of 0.32/1000 youths [56]. The high percentage of T2D subtype among incident diabetes diagnoses in our data is therefore notable, comprising almost half or more of all diagnoses during all variant eras. Possible causes of the high proportion of T2D diagnoses may include a shift in subtype incidence due to pandemic‐related lifestyle changes, as well as increased screening and diagnosis in the pediatric population due to interaction with the healthcare system. Prior to the pandemic, epidemiologic trends have shown increasing rates of T2D in pediatric patients [56–59], which may be exacerbated by worsening rates of obesity and sedentary lifestyle during the pandemic era [5]. The observed shift in subtype predominance from T1D to T2D among pediatric patients is particularly alarming, underscoring not only the urgent need for aggressive management of modifiable behavioral risk factors but also the profound public health implications of a growing pediatric T2D burden that could strain health systems and increase the long‐term risk of serious diabetes‐related complications.

Overall, our study did not show an increase in the risk of incident diabetes in pediatric populations compared to COVID‐19‐negative and ARI control groups during most of the viral variant eras, or for either T1D or T2D subtypes. This is in direct contrast to findings from other studies, which show an increased risk of T1D [18, 21, 25] and T2D [16] after COVID‐19, and differences may potentially be accounted for by several factors. Prior studies defined the initial cohort without diabetes, and the primary outcome of incident diabetes based on diagnosis code; our study cohort selection criteria and outcome definition relied on a combination of diagnosis codes, laboratory criteria, and data quality metrics. Additionally, while most studies matched or adjusted for demographic covariates, we adjusted for BMI percentile and interaction with data partners to account for population differences by health care facility. The data source for several of the studies were claimed, which do not have the granularity of EHR data [18, 25]. Additionally, prior studies utilized data from early in the pandemic, which was a period where non‐COVID‐19 health care utilization was lower as resources were directed toward COVID‐19‐related care, and there was more potential for diagnoses of conditions such as diabetes in individuals who presented with COVID‐19. It was somewhat surprising that our findings differed from those reported by Miller et al. [16] of increased T2D at 1, 3, and 6 months compared to other non‐COVID‐19 respiratory infections, and which also utilized a large database over a similar time frame. Possible differences in risk [16] and incidence rates based on community‐based populations [59] may be partially explained by data source, as individuals in the N3C Enclave are extracted based on age‐ and gender‐matching of patients with COVID‐19 negative tests with COVID‐19 positive cases, and may be more representative of the demographics of the COVID‐19 positive population. However, given the large samples and geographic distribution of N3C sites [60], our findings are fairly representative of the pediatric population receiving care within the pandemic era. Additional study or data parameters, such as outcome definition or cohort balancing methods, and percent missingness of important modifiers such as BMI [16], may contribute to the contrasting findings. Additionally, the COVID‐19‐negative cohort had a higher rate of hospitalization and corticosteroid use, which may be associated with a greater risk of developing diabetes. However, results were similar in the ARI group, which was more comparable to the COVID‐19 group with regard to hospitalization. Overall, it remains unclear whether there is an increased risk of incident diabetes in pediatric patients after COVID‐19.

There were several limitations in our study. Unmeasured confounding from factors that are not routinely recorded in EHR data, such as socio–economic status, environmental exposures, and behaviors related to diet and physical activity levels were not included in our analysis, which may have a significant impact on lifestyle‐related diseases like T2D. In addition, the viral variant era was defined by a time period rather than viral variant laboratory data, which was not routinely available for most COVID‐19 cases. While individuals within a time period may have been infected with a different variant, this is unlikely to systemically bias results. Incident diabetes outcomes were defined by diagnosis code and HbA1c criteria, as we could not consistently identify laboratory values associated with oral glucose tolerance testing. The N3C also includes data from contributing sites across the US, many of which are large tertiary care health centers, and which may represent a population with a higher burden of illness. This is reflected in the higher rates of hospitalization and corticosteroid use in the COVID‐19 negative group; however, we sought to mitigate potential differences in illness severity by using an ARI control group. While we were able to detect trends in incidence rates over viral variants, the patient population in the N3C may not reflect incidence in the general population. Additionally, there may be issues with ascertainment of COVID‐19‐negative controls in data from later in the pandemic, when home testing for COVID‐19 was widespread and infections may be poorly documented in EHR data. However, our findings indicate no clinical difference in risk between COVID‐19‐negative and ARI control groups even early in the pandemic, when the ascertainment of COVID‐19 status was less challenging.

## 4. Conclusion

Overall, we found that for most variant eras, there was no difference in the risk of incident diabetes in pediatric patients after COVID‐19 compared to patients in COVID‐19‐negative or ARI control groups. Incident diabetes cases were predominantly T2D subtype, and rates followed a U‐shaped curve, with the highest incidence in the ancestral variant period. Understanding the trajectory of T2D incidence in the pandemic era, and aggressive management of lifestyle and behavioral factors are extremely important to prevent diabetes and related morbidity in the US pediatric population.

## Ethics Statement

The N3C data transfer to NCATS is performed under a Johns Hopkins University Reliance Protocol # IRB00249128 or individual site agreements with NIH. The N3C Data Enclave is managed under the authority of the NIH; information can be found at https://ncats.nih.gov/n3c/resources.

## Disclosure

The N3C Publication committee confirmed that this manuscript, MSID: 2329.516, is in accordance with N3C data use and attribution policies; however, this content is solely the responsibility of the authors and does not necessarily represent the official views of the National Institutes of Health or the N3C program. The following institutions whose data is released or pending: Available: Advocate Health Care Network—UL1TR002389: The Institute for Translational Medicine (ITM) • Aurora Health Care Inc—UL1TR002373: Wisconsin Network For Health Research • Boston University Medical Campus—UL1TR001430: Boston University Clinical and Translational Science Institute • Brown University—U54GM115677: Advance Clinical Translational Research (Advance‐CTR) • Carilion Clinic—UL1TR003015: iTHRIV Integrated Translational health Research Institute of Virginia • Case Western Reserve University—UL1TR002548: The Clinical & Translational Science Collaborative of Cleveland (CTSC) • Charleston Area Medical Center—U54GM104942: West Virginia Clinical and Translational Science Institute (WVCTSI) • Children’s Hospital Colorado—UL1TR002535: Colorado Clinical and Translational Sciences Institute • Columbia University Irving Medical Center—UL1TR001873: Irving Institute for Clinical and Translational Research • Dartmouth College—None (Voluntary) Duke University—UL1TR002553: Duke Clinical and Translational Science Institute • George Washington Children’s Research Institute—UL1TR001876: Clinical and Translational Science Institute at Children’s National (CTSA‐CN) • George Washington University—UL1TR001876: Clinical and Translational Science Institute at Children’s National (CTSA‐CN) • Harvard Medical School—UL1TR002541: Harvard Catalyst • Indiana University School of Medicine—UL1TR002529: Indiana Clinical and Translational Science Institute • Johns Hopkins University—UL1TR003098: Johns Hopkins Institute for Clinical and Translational Research • Louisiana Public Health Institute—None (Voluntary) • Loyola Medicine—Loyola University Medical Center • Loyola University Medical Center—UL1TR002389: The Institute for Translational Medicine (ITM) • Maine Medical Center—U54GM115516: Northern New England Clinical & Translational Research (NNE‐CTR) Network • Mary Hitchcock Memorial Hospital & Dartmouth Hitchcock Clinic—None (Voluntary) • Massachusetts General Brigham—UL1TR002541: Harvard Catalyst • Mayo Clinic Rochester—UL1TR002377: Mayo Clinic Center for Clinical and Translational Science (CCaTS) • Medical University of South Carolina—UL1TR001450: South Carolina Clinical & Translational Research Institute (SCTR) • MITRE Corporation—None (Voluntary) • Montefiore Medical Center—UL1TR002556: Institute for Clinical and Translational Research at Einstein and Montefiore • Nemours—U54GM104941: Delaware CTR ACCEL Program • NorthShore University HealthSystem—UL1TR002389: The Institute for Translational Medicine (ITM) • Northwestern University at Chicago—UL1TR001422: Northwestern University Clinical and Translational Science Institute (NUCATS) • OCHIN—INV‐018455: Bill and Melinda Gates Foundation grant to Sage Bionetworks • Oregon Health & Science University—UL1TR002369: Oregon Clinical and Translational Research Institute • Penn State Health Milton S. Hershey Medical Center—UL1TR002014: Penn State Clinical and Translational Science Institute • Rush University Medical Center—UL1TR002389: The Institute for Translational Medicine (ITM) • Rutgers, The State University of New Jersey—UL1TR003017: New Jersey Alliance for Clinical and Translational Science • Stony Brook University—U24TR002306 • The Alliance at the University of Puerto Rico, Medical Sciences Campus—U54GM133807: Hispanic Alliance for Clinical and Translational Research (The Alliance) • The Ohio State University—UL1TR002733: Center for Clinical and Translational Science • The State University of New York at Buffalo—UL1TR001412: Clinical and Translational Science Institute • The University of Chicago—UL1TR002389: The Institute for Translational Medicine (ITM) • The University of Iowa—UL1TR002537: Institute for Clinical and Translational Science • The University of Miami Leonard M. Miller School of Medicine—UL1TR002736: University of Miami Clinical and Translational Science Institute • The University of Michigan at Ann Arbor—UL1TR002240: Michigan Institute for Clinical and Health Research • The University of Texas Health Science Center at Houston—UL1TR003167: Center for Clinical and Translational Sciences (CCTS) • The University of Texas Medical Branch at Galveston—UL1TR001439: The Institute for Translational Sciences • The University of Utah—UL1TR002538: Uhealth Center for Clinical and Translational Science • Tufts Medical Center—UL1TR002544: Tufts Clinical and Translational Science Institute • Tulane University—UL1TR003096: Center for Clinical and Translational Science • The Queens Medical Center—None (Voluntary) • University Medical Center New Orleans—U54GM104940: Louisiana Clinical and Translational Science (LA CaTS) Center • University of Alabama at Birmingham—UL1TR003096: Center for Clinical and Translational Science • University of Arkansas for Medical Sciences—UL1TR003107: UAMS Translational Research Institute • University of Cincinnati—UL1TR001425: Center for Clinical and Translational Science and Training • University of Colorado Denver, Anschutz Medical Campus—UL1TR002535: Colorado Clinical and Translational Sciences Institute • University of Illinois at Chicago—UL1TR002003: UIC Center for Clinical and Translational Science • University of Kansas Medical Center—UL1TR002366: Frontiers: University of Kansas Clinical and Translational Science Institute • University of Kentucky—UL1TR001998: UK Center for Clinical and Translational Science • University of Massachusetts Medical School Worcester—UL1TR001453: The UMass Center for Clinical and Translational Science (UMCCTS) • University Medical Center of Southern Nevada—None (voluntary) • University of Minnesota—UL1TR002494: Clinical and Translational Science Institute • University of Mississippi Medical Center—U54GM115428: Mississippi Center for Clinical and Translational Research (CCTR) • University of Nebraska Medical Center—U54GM115458: Great Plains IDeA‐Clinical & Translational Research • University of North Carolina at Chapel Hill—UL1TR002489: North Carolina Translational and Clinical Science Institute • University of Oklahoma Health Sciences Center—U54GM104938: Oklahoma Clinical and Translational Science Institute (OCTSI) • University of Pittsburgh—UL1TR001857: The Clinical and Translational Science Institute (CTSI) • University of Pennsylvania—UL1TR001878: Institute for Translational Medicine and Therapeutics • University of Rochester—UL1TR002001: UR Clinical & Translational Science Institute • University of Southern California—UL1TR001855: The Southern California Clinical and Translational Science Institute (SC CTSI) • University of Vermont—U54GM115516: Northern New England Clinical & Translational Research (NNE‐CTR) Network • University of Virginia—UL1TR003015: iTHRIV Integrated Translational health Research Institute of Virginia • University of Washington—UL1TR002319: Institute of Translational Health Sciences • University of Wisconsin‐Madison—UL1TR002373: UW Institute for Clinical and Translational Research • Vanderbilt University Medical Center—UL1TR002243: Vanderbilt Institute for Clinical and Translational Research • Virginia Commonwealth University—UL1TR002649: C. Kenneth and Dianne Wright Center for Clinical and Translational Research • Wake Forest University Health Sciences—UL1TR001420: Wake Forest Clinical and Translational Science Institute • Washington University in St. Louis—UL1TR002345: Institute of Clinical and Translational Sciences • Weill Medical College of Cornell University—UL1TR002384: Weill Cornell Medicine Clinical and Translational Science Center • West Virginia University—U54GM104942: West Virginia Clinical and Translational Science Institute (WVCTSI) Submitted: Icahn School of Medicine at Mount Sinai—UL1TR001433: ConduITS Institute for Translational Sciences • The University of Texas Health Science Center at Tyler—UL1TR003167: Center for Clinical and Translational Sciences (CCTS) • University of California, Davis—UL1TR001860: UCDavis Health Clinical and Translational Science Center • University of California, Irvine—UL1TR001414: The UC Irvine Institute for Clinical and Translational Science (ICTS) • University of California, Los Angeles—UL1TR001881: UCLA Clinical Translational Science Institute • University of California, San Diego—UL1TR001442: Altman Clinical and Translational Research Institute • University of California, San Francisco—UL1TR001872: UCSF Clinical and Translational Science Institute NYU Langone Health Clinical Science Core, Data Resource Core, and PASC Biorepository Core—OTA‐21‐015 A: Post‐Acute Sequelae of SARS‐CoV‐2 Infection Initiative (RECOVER) Pending: Arkansas Children’s Hospital—UL1TR003107: UAMS Translational Research Institute • Baylor College of Medicine—None (Voluntary) • Children’s Hospital of Philadelphia—UL1TR001878: Institute for Translational Medicine and Therapeutics • Cincinnati Children’s Hospital Medical Center—UL1TR001425: Center for Clinical and Translational Science and Training • Emory University—UL1TR002378: Georgia Clinical and Translational Science Alliance • HonorHealth—None (Voluntary) • Loyola University Chicago—UL1TR002389: The Institute for Translational Medicine (ITM) • Medical College of Wisconsin—UL1TR001436: Clinical and Translational Science Institute of Southeast Wisconsin • MedStar Health Research Institute—None (Voluntary) • Georgetown University—UL1TR001409: The Georgetown‐Howard Universities Center for Clinical and Translational Science (GHUCCTS) • MetroHealth—None (Voluntary) • Montana State University—U54GM115371: American Indian/Alaska Native CTR • NYU Langone Medical Center—UL1TR001445: Langone Health’s Clinical and Translational Science Institute • Ochsner Medical Center—U54GM104940: Louisiana Clinical and Translational Science (LA CaTS) Center • Regenstrief Institute—UL1TR002529: Indiana Clinical and Translational Science Institute • Sanford Research—None (Voluntary) • Stanford University—UL1TR003142: Spectrum: The Stanford Center for Clinical and Translational Research and Education • The Rockefeller University—UL1TR001866: Center for Clinical and Translational Science • The Scripps Research Institute—UL1TR002550: Scripps Research Translational Institute • University of Florida—UL1TR001427: UF Clinical and Translational Science Institute • University of New Mexico Health Sciences Center—UL1TR001449: University of New Mexico Clinical and Translational Science Center • University of Texas Health Science Center at San Antonio—UL1TR002645: Institute for Integration of Medicine and Science • Yale New Haven Hospital—UL1TR001863: Yale Center for Clinical Investigation.

## Conflicts of Interest

John B. Buse has received salary support from clinical trial contracts to his employer by Corcept, Dexcom, GentiBio, and Novo Nordisk; he is a consultant with personal compensation from Aardvark Therapeutics, Altimmune, Alveus Therapeutics, Amgen, Antag, Aqua Medical, AstraZeneca, Boehringer‐Ingelheim, Corcept Therapeutics, Dexcom, Eli Lilly, embecta, General Medicines Inc, GentiBio, Insulet, Kayothera, Metsera, MiniMed, Novo Nordisk, Recordati, Sparrow Pharmaceuticals, Tandem, Vertex, vTv Therapeutics, and Zealand; he has stock/options in Glyscend, Mellitus Health, Metsera, Pendulum Therapeutics, Praetego, and Stability Health. Til Stürmer owns stock in Novartis, Roche, and Novo Nordisk; he does not accept personal compensation of any kind from any pharmaceutical company.

## Author Contributions

Authorship was determined using the ICMJE recommendations. Margaret A. Hall cleaned and analyzed data. Talia Wiggen cleaned and analyzed data. Steven G. Johnson cleaned and analyzed data. Lindsey E. Turner cleaned and analyzed data. Jared D. Huling provided statistical expertise and reviewed/edited the manuscript. Kenneth J. Wilkins provided statistical expertise and reviewed/edited the manuscript. Carolyn T. Bramante reviewed/edited the manuscript. Hsin‐Chieh Yeh provided epidemiology expertise and reviewed/edited the manuscript. Til Stürmer provided epidemiology expertise and reviewed/edited the manuscript. John B. Buse provided clinical expertise and reviewed/edited the manuscript. Jane Reusch provided clinical expertise and reviewed/edited the manuscript. Zachary Butzin‐Dozier provided statistical expertise and reviewed/edited the manuscript. Rachel Wong is the guarantor of this work and, as such, had full access to all the data in the study and takes responsibility for the integrity of the data and the accuracy of the data analysis.

## Funding

Rachel Wong, Jane Reusch, Steven G. Johnson, and Hsin‐Chieh Yeh receive funding from (Grant 3R01DK130351‐ 02S1) the National Institutes of Health (NIH). Carolyn T. Bramante is funded by the National Institute of Digestive and Diabetes and Kidney Diseases (Grant K23DK124654). Til Stürmer receives investigator‐initiated research funding and support as principal investigator (Grant R01AG056479) from the National Institute on Aging (NIA), and as coinvestigator (Grant R01CA277756) from the National Cancer Institute, National Institutes of Health (NIH). He also receives salary support as director of Comparative Effectiveness Research (CER), NC TraCS Institute, UNC Clinical and Translational Science Award (Grant UM1TR004406), codirector of the Human Studies Consultation Core, NC Diabetes Research Center (Grant P30DK124723), National Institute of Diabetes and Digestive and Kidney Diseases (NIDDK), the Center for Pharmacoepidemiology (current members: GlaxoSmithKline, UCB BioSciences, Takeda, AbbVie, Boehringer Ingelheim, Astellas, and Sarepta), and from a generous contribution from Dr. Nancy A. Dreyer to the Department of Epidemiology, University of North Carolina at Chapel Hill for nonrelated work. John B. Buse was supported by grants from the NIH (Grants UL1TR002489, UM1TR004406, and P30DK124723).

## Supporting Information

Additional supporting information can be found online in the Supporting Information section.

## Supporting information


**Supporting Information** Text Box 1. Description of pediatric body mass index categories. Table S1. Baseline characteristics of COVID‐19‐positive, COVID‐19‐negative, and ARI patients in the Ancestral variant era. Table S2. Baseline characteristics of COVID‐19‐positive, COVID‐19‐negative, and ARI patients in the Alpha variant era. Table S3. Baseline characteristics of COVID‐19‐positive, COVID‐19‐negative, and ARI patients in the Delta variant era. Table S4. Baseline characteristics of COVID‐19‐positive, COVID‐19‐negative, and ARI patients in the Omicron variant era. Table S5. Number at risk by viral variant for COVID‐19‐positive, COVID‐19‐negative, and ARI patients. Table S6. Number at risk by viral variant and hospitalization for COVID‐19‐positive, COVID‐19‐negative, and ARI patients. Table S7. Weighted count of censoring events by viral variant for COVID‐19‐positive, COVID‐19‐negative, and ARI patients. Table S8. Total uncensored and unweighted cases of new diabetes across all groups and proportion of cases by variant. Figure S9. Cumulative incidence of death in pediatric patients after COVID‐19 compared to COVID‐negative and ARI controls by COVID variant and time period. Figure S10. Cumulative incidence of diabetes in hospitalized and nonhospitalized pediatric patients after COVID‐19, compared to COVID‐negative and ARI controls by COVID variant and time period. Table S11. Cumulative incidence of diabetes by viral variant for COVID‐19‐positive, COVID‐19‐negative and ARI patients.

## Data Availability

The data that support the findings of this study are openly available in the National COVID Cohort Collaborative at https://covid.cd2h.org/.
