# Supplementary material for: The Effect of COVID‐19 on Incident Diabetes in Pediatric Patients: Findings From the National COVID‐19 Cohort Collaborative (N3C)
Source: Pediatr Diabetes. 2025 Dec 5;2025:3545727. doi: 10.1155/pedi/3545727 (PMC12707300; doi:10.1155/pedi/3545727)
Supplement: Supplementary file 1 — Supporting Information Text Box 1. Description of pediatric body mass index categories. Table S1. Baseline characteristics of COVID‐19‐positive, COVID‐19‐negative, and ARI patients in the Ancestral variant era. Table S2. Baseline characteristics of COVID‐19‐positive, COVID‐19‐negative, and ARI patients in the Alpha variant era. Table S3. Baseline characteristics of COVID‐19‐positive, COVID‐19‐negative, and ARI patients in the Delta variant era. Table S4. Baseline characteristics of COVID‐19‐positive, COVID‐19‐negative, and ARI patients in the Omicron variant era. Table S5. Number at risk by viral variant for COVID‐19‐positive, COVID‐19‐negative, and ARI patients. Table S6. Number at risk by viral variant and hospitalization for COVID‐19‐positive, COVID‐19‐negative, and ARI patients. Table S7. Weighted count of censoring events by viral variant for COVID‐19‐positive, COVID‐19‐negative, and ARI patients. Table S8. Total uncensored and unweighted cases of new diabetes across all groups and proportion of cases by variant. Figure S9. Cumulative incidence of death in pediatric patients after COVID‐19 compared to COVID‐negative and ARI controls by COVID variant and time period. Figure S10. Cumulative incidence of diabetes in hospitalized and nonhospitalized pediatric patients after COVID‐19, compared to COVID‐negative and ARI controls by COVID variant and time period. Table S11. Cumulative incidence of diabetes by viral variant for COVID‐19‐positive, COVID‐19‐negative and ARI patients. [file PEDI-2025-3545727-s001.zip › Supplementary Materials_1.docx]

**Supplementary Index Table of Contents**

**Text Box 1.** Description of Pediatric Body Mass Index Categories…………………….....2

**Table S1.** Baseline characteristics of COVID-19 positive, COVID-19 negative and ARI

patients in the Ancestral variant era………………………………………………………..…3

**Table S2.** Baseline characteristics of COVID-19 positive, COVID-19 negative and ARI

patients in the Alpha variant era ………………………………………………………...……5

**Table S3.** Baseline characteristics of COVID-19 positive, COVID-19 negative and ARI

patients in the Delta variant era …………………………………………………………...….7

**Table S4.** Baseline characteristics of COVID-19 positive, COVID-19 negative and ARI

patients in the Omicron variant era ………………………………………………………...…9

**Table S5.** Number at risk by viral variant for COVID-19 positive, COVID-19

negative and ARI patients…………………………………………………………….…..…..11

**Table S6.** Number at risk by viral variant and hospitalization for COVID-19 positive,

COVID-19 negative and ARI patients…………...………………………….……………….12

**Table S7.** Weighted count of censoring events by viral variant for COVID-19 positive, COVID-19 negative and ARI patients……..…..…………………………………………….13

**Table S8.** Total uncensored and unweighted cases of new diabetes across all groups and proportion of cases by variant……...………………………………………..………….14

**Figure S9.** Cumulative incidence of death in pediatric patients after COVID-19 compared to COVID negative and ARI controls by COVID variant and time period..….15

**Figure S10.** Cumulative incidence of diabetes in hospitalized and non-hospitalized

pediatric patients after COVID-19 compared to COVID negative and ARI controls by COVID variant and time period………………………………………………………………16

**Table S11.** Cumulative incidence of diabetes by viral variant for COVID-19 positive,

COVID-19 negative and ARI patients……………..……...…………………………..….….17

**Text Box 1.** Description of Pediatric Body Mass Index Categories

| **Body Mass Index (BMI) Categories Based on Percentile for Age and Sex:**   - Underweight: BMI < 5th percentile - Normal Weight: BMI >= 5th to < 85th percentile - Overweight: BMI >= 85th to < 95th percentile - Class 1 Obesity: BMI >= 95th to 120% of the 95th percentile - Class 2 Obesity: BMI >= 120% to < 140% of the 95th percentile - Class 3 Obesity: BMI >= 140% of the 95th percentile   *Source:* Center for Disease Control and Prevention, Child and Teen BMI Categories [*https://www.cdc.gov/bmi/child-teen-calculator/bmi-categories.html*](https://www.cdc.gov/bmi/child-teen-calculator/bmi-categories.html) |
| --- |

**Table S1.** Baseline characteristics of COVID-19 positive, COVID-19 negative and ARI

patients in the Ancestral variant era

|  | **Unweighted** | | | | **Weighted** | | | |
| --- | --- | --- | --- | --- | --- | --- | --- | --- |
|  | **COVID Positive (n=16,926)** | **COVID Negative (n=96,138)** | **ARI (n=34,352)** | **SMD** | **COVID Positive** | **COVID Negative** | **ARI** | **SMD** |
|  |  |  |  |  |  |  |  |  |
| Gender | n (%) | n (%) | n (%) |  | % | % | % |  |
| Female | 8,454 (49.9) | 45,848 (47.7) | 17,429 (50.7) | 0.042 | 48.5 | 48.7 | 48.7 | 0.011 |
| Male | 8,470 (50.0) | 50,270 (52.3) | 16,921 (49.3) |  | 51.5 | 51.3 | 51.3 |  |
| Age |  |  |  |  |  |  |  |  |
| <1 | 1,180 (7.0) | 11,159 (11.6) | 6,162 (17.9) | 0.257 | 12 | 12.5 | 12.5 | 0.017 |
| 1 to 4 | 3,315 (19.6) | 21,153 (22.0) | 7,623 (22.2) |  | 21.5 | 21.8 | 21.7 |  |
| 5 to 9 | 3,694 (21.8) | 19,407 (20.2) | 7,403 (21.6) |  | 20.4 | 20.7 | 20.8 |  |
| 10 to 17 | 8,737 (51.6) | 44,419 (46.2) | 13,164 (38.3) |  | 46.1 | 45 | 45 |  |
| Race/Ethnicity | | | | | | | | |
| American Indian or Alaska Native Non-Hispanic | 111 (0.7) | 510 (0.5) | 167 (0.5) | 0.44 | 0.4 | 0.5 | 0.5 | 0.02 |
| Asian Non-Hispanic | 439 (2.6) | 2,960 (3.1) | 951 (2.8) |  | 3.1 | 3 | 2.9 |  |
| Black or African American Non-Hispanic | 2,556 (15.1) | 13,042 (13.6) | 5,133 (14.9) |  | 14.2 | 14.1 | 14 |  |
| Hispanic or Latino Any Race | 7,386 (43.6) | 18,047 (18.8) | 6,439 (18.7) |  | 22.1 | 21.7 | 21.7 |  |
| Native Hawaiian or Other Pacific Islander Non-Hispanic | 45 (0.3) | 166 (0.2) | 51 (0.1) |  | 0.1 | 0.2 | 0.1 |  |
| White Non-Hispanic | 5,141 (30.4) | 52,473 (54.6) | 19,139 (55.7) |  | 51.3 | 51.9 | 52.3 |  |
| Other Non-Hispanic | 103 (0.6) | 939 (1.0) | 196 (0.6) |  | 0.8 | 0.8 | 0.8 |  |
| Unknown | 1,145 (6.8) | 8,001 (8.3) | 2,276 (6.6) |  | 7.9 | 7.8 | 7.6 |  |
| BMI Category (calculated on those with age greater than 2 years) | | | | | | | | |
| 0. Age less than 2 | 2,132 (12.6) | 19,335 (20.1) | 9,158 (26.7) | 0.294 | 20 | 20.7 | 20.7 | 0.023 |
| 0. Missing | 5,502 (32.5) | 29,306 (30.5) | 11,726 (34.1) |  | 31.8 | 31.3 | 31.5 |  |
| 1. Underweight | 305 (1.8) | 2,588 (2.7) | 479 (1.4) |  | 2.2 | 2.3 | 2.3 |  |
| 2. Normal weight | 4,717 (27.9) | 26,986 (28.1) | 7,227 (21.0) |  | 27.1 | 26.6 | 26.6 |  |
| 2. Overweight | 1,671 (9.9) | 7,528 (7.8) | 2,400 (7.0) |  | 7.7 | 7.9 | 7.9 |  |
| 3. Class I Obesity | 1,574 (9.3) | 6,502 (6.8) | 2,130 (6.2) |  | 6.9 | 7 | 6.9 |  |
| 4. Class II Obesity | 618 (3.7) | 2,405 (2.5) | 785 (2.3) |  | 2.7 | 2.6 | 2.6 |  |
| 5. Class III Obesity | 406 (2.4) | 1,475 (1.5) | 447 (1.3) |  | 1.6 | 1.6 | 1.6 |  |
| Treatment | | | | | | | | |
| Remdesivir | <20 | 0 (0.0) | 0 (0.0) | 0.028 | <20 | 0 | 0 | 0.029 |
| Corticosteroids | 617 (3.6) | 17,040 (17.7) | 2,101 (6.1) | 0.316 | 3.7 | 17.2 | 7.1 | 0.305 |
| Disease Severity | | | | | | | | |
| Hospitalization | 579 (3.4) | 12,621 (13.1) | 767 (2.2) | 0.283 | 4.2 | 12.5 | 3 | 0.243 |

**Table S2.** Baseline characteristics of COVID-19 positive, COVID-19 negative and ARI

patients in the Alpha variant era

|  | **Unweighted** | | | | **Weighted** | | | |
| --- | --- | --- | --- | --- | --- | --- | --- | --- |
|  | **COVID Positive (n=68,826)** | **COVID Negative (n=223,276)** | **ARI (n=52,692)** | **SMD** | **COVID Positive** | **COVID Negative** | **ARI** | **SMD** |
|  |  |  |  |  |  |  |  |  |
| Gender | n (%) | n (%) | n (%) |  | % | % | % |  |
| Female | 34,115 (49.6) | 107,040 (47.9) | 26,307 (49.9) | 0.027 | 48.7 | 48.6 | 48.8 | 0.008 |
| Male | 34,703 (50.4) | 116,205 (52.0) | 26,379 (50.1) |  | 51.3 | 51.4 | 51.2 |  |
| Age |  |  |  |  |  |  |  |  |
| <1 | 3,455 (5.0) | 24,917 (11.2) | 11,208 (21.3) | 0.397 | 11.5 | 11.5 | 11.5 | 0.003 |
| 1 to 4 | 12,309 (17.9) | 49,807 (22.3) | 13,258 (25.2) |  | 21.7 | 21.8 | 21.9 |  |
| 5 to 9 | 15,012 (21.8) | 47,243 (21.2) | 10,009 (19.0) |  | 20.9 | 20.9 | 20.9 |  |
| 10 to 17 | 38,050 (55.3) | 101,309 (45.4) | 18,217 (34.6) |  | 45.9 | 45.7 | 45.7 |  |
| Race/Ethnicity |  |  |  |  |  |  |  |  |
| American Indian or Alaska Native Non-Hispanic | 383 (0.6) | 1,077 (0.5) | 216 (0.4) | 0.146 | 0.5 | 0.5 | 0.5 | 0.011 |
| Asian Non-Hispanic | 1,863 (2.7) | 7,273 (3.3) | 1,341 (2.5) |  | 3.1 | 3 | 3.1 |  |
| Black or African American Non-Hispanic | 9,360 (13.6) | 31,944 (14.3) | 7,748 (14.7) |  | 14.3 | 14.2 | 14 |  |
| Hispanic or Latino Any Race | 17,211 (25.0) | 41,748 (18.7) | 9,529 (18.1) |  | 20 | 19.9 | 20 |  |
| Native Hawaiian or Other Pacific Islander Non-Hispanic | 86 (0.1) | 322 (0.1) | 55 (0.1) |  | 0.1 | 0.1 | 0.1 |  |
| White Non-Hispanic | 34,503 (50.1) | 117,899 (52.8) | 29,490 (56.0) |  | 52.4 | 52.7 | 52.9 |  |
| Other Non-Hispanic | 426 (0.6) | 1,915 (0.9) | 432 (0.8) |  | 0.8 | 0.8 | 0.8 |  |
| Unknown | 4,994 (7.3) | 21,098 (9.4) | 3,881 (7.4) |  | 8.8 | 8.7 | 8.6 |  |
| BMI Category (calculated on those with age greater than 2 years) | | | | | | | | |
| 0. Age less than 2 | 7,047 (10.2) | 44,931 (20.1) | 17,747 (33.7) | 0.403 | 20.3 | 20.2 | 20.3 | 0.009 |
| 0. Missing | 25,120 (36.5) | 71,082 (31.8) | 14,767 (28.0) |  | 32.2 | 32.2 | 32 |  |
| 1. Underweight | 1,130 (1.6) | 5,343 (2.4) | 659 (1.3) |  | 2.1 | 2.1 | 2.1 |  |
| 2. Normal weight | 19,092 (27.7) | 59,656 (26.7) | 10,702 (20.3) |  | 25.8 | 25.9 | 26 |  |
| 2. Overweight | 6,364 (9.2) | 17,408 (7.8) | 3,448 (6.5) |  | 7.9 | 7.9 | 8 |  |
| 3. Class I Obesity | 6,003 (8.7) | 15,350 (6.9) | 3,254 (6.2) |  | 7.2 | 7.1 | 7.2 |  |
| 4. Class II Obesity | 2,477 (3.6) | 5,883 (2.6) | 1,284 (2.4) |  | 2.8 | 2.8 | 2.8 |  |
| 5. Class III Obesity | 1,591 (2.3) | 3,607 (1.6) | 831 (1.6) |  | 1.7 | 1.7 | 1.7 |  |
| Treatment |  |  |  |  |  |  |  |  |
| Paxlovid | 0 (0.0) | 0 (0.0) | <20 | 0.004 | 0 | 0 | <20 | 0.005 |
| Remdesivir | 93 (0.1) | 0 (0.0) | 0 (0.0) | 0.035 | 0.1 | 0 | 0 | 0.036 |
| Corticosteroids | 2,040 (3.0) | 27,696 (12.4) | 3,423 (6.5) | 0.243 | 3.1 | 12.3 | 6.5 | 0.236 |
| Disease Severity |  |  |  |  |  |  |  |  |
| Hospitalization | 1,362 (2.0) | 19,181 (8.6) | 858 (1.6) | 0.215 | 2.6 | 8.4 | 1.8 | 0.207 |

**Table S3.** Baseline characteristics of COVID-19 positive, COVID-19 negative and ARI

patients in the Delta variant era

|  | **Unweighted** | | | | **Weighted** | | | |
| --- | --- | --- | --- | --- | --- | --- | --- | --- |
|  | **COVID Positive (n=66,525)** | **COVID Negative (n=174,915)** | **ARI (n=50,003)** | **SMD** | **COVID Positive** | **COVID Negative** | **ARI** | **SMD** |
|  |  |  |  |  |  |  |  |  |
| Gender | n (%) | n (%) | n (%) |  | % | % | % |  |
| Female | 33,092 (49.7) | 84,176 (48.1) | 24,180 (48.4) | 0.023 | 48.8 | 48.5 | 48.7 | 0.008 |
| Male | 33,426 (50.2) | 90,693 (51.8) | 25,811 (51.6) |  | 51.2 | 51.5 | 51.3 |  |
| Age |  |  |  |  |  |  |  |  |
| <1 | 3,408 (5.1) | 20,060 (11.5) | 9,774 (19.5) | 0.425 | 11.5 | 11.4 | 11.4 | 0.003 |
| 1 to 4 | 12,253 (18.4) | 46,573 (26.6) | 16,090 (32.2) |  | 25.7 | 25.7 | 25.7 |  |
| 5 to 9 | 18,611 (28.0) | 39,782 (22.7) | 9,383 (18.8) |  | 23.2 | 23.2 | 23.1 |  |
| 10 to 17 | 32,253 (48.5) | 68,500 (39.2) | 14,756 (29.5) |  | 39.7 | 39.6 | 39.7 |  |
| Race/Ethnicity | | | | | | | | |
| American Indian or Alaska Native Non-Hispanic | 407 (0.6) | 1,105 (0.6) | 196 (0.4) | 0.183 | 0.6 | 0.6 | 0.6 | 0.015 |
| Asian Non-Hispanic | 1,290 (1.9) | 7,019 (4.0) | 1,789 (3.6) |  | 3.4 | 3.5 | 3.4 |  |
| Black or African American Non-Hispanic | 9,960 (15.0) | 25,333 (14.5) | 7,896 (15.8) |  | 15.1 | 14.8 | 14.6 |  |
| Hispanic or Latino Any Race | 10,611 (16.0) | 36,254 (20.7) | 10,915 (21.8) |  | 19.9 | 19.8 | 19.8 |  |
| Native Hawaiian or Other Pacific Islander Non-Hispanic | 78 (0.1) | 259 (0.1) | 69 (0.1) |  | 0.1 | 0.1 | 0.1 |  |
| White Non-Hispanic | 38,911 (58.5) | 85,143 (48.7) | 23,961 (47.9) |  | 50.2 | 50.8 | 51.1 |  |
| Other Non-Hispanic | 524 (0.8) | 1,536 (0.9) | 457 (0.9) |  | 0.9 | 0.9 | 0.9 |  |
| Unknown | 4,744 (7.1) | 18,266 (10.4) | 4,720 (9.4) |  | 9.8 | 9.5 | 9.5 |  |
| BMI Category (calculated on those with age greater than 2 years) | | | | | | | | |
| 0. Age less than 2 | 6,681 (10.0) | 38,487 (22.0) | 17,516 (35.0) | 0.455 | 21.6 | 21.5 | 21.5 | 0.007 |
| 0. Missing | 25,590 (38.5) | 55,621 (31.8) | 9,870 (19.7) |  | 31.2 | 31.3 | 31.1 |  |
| 1. Underweight | 1,212 (1.8) | 4,017 (2.3) | 860 (1.7) |  | 2.1 | 2.1 | 2.1 |  |
| 2. Normal weight | 17,590 (26.4) | 43,467 (24.9) | 11,715 (23.4) |  | 24.9 | 25 | 25 |  |
| 2. Overweight | 5,747 (8.6) | 12,792 (7.3) | 3,737 (7.5) |  | 7.7 | 7.7 | 7.7 |  |
| 3. Class I Obesity | 5,472 (8.2) | 12,248 (7.0) | 3,790 (7.6) |  | 7.4 | 7.4 | 7.4 |  |
| 4. Class II Obesity | 2,504 (3.8) | 5,078 (2.9) | 1,530 (3.1) |  | 3.1 | 3.1 | 3.2 |  |
| 5. Class III Obesity | 1,727 (2.6) | 3,188 (1.8) | 984 (2.0) |  | 2 | 2 | 2 |  |
| Treatment | | | | | | | | |
| Remdesivir | 172 (0.3) | 0 (0.0) | 0 (0.0) | 0.048 | 0.3 | 0 | 0 | 0.051 |
| Corticosteroids | 2,806 (4.2) | 15,688 (9.0) | 4,157 (8.3) | 0.128 | 4.1 | 9.4 | 8 | 0.143 |
| Disease Severity | | | | | | | | |
| Hospitalization | 1,420 (2.1) | 12,312 (7.0) | 1,220 (2.4) | 0.158 | 2.7 | 7.1 | 2.1 | 0.161 |

**Table S4.** Baseline characteristics of COVID-19 positive, COVID-19 negative and ARI

patients in the Omicron variant era

|  | **Unweighted** | | | | **Weighted** | | | |
| --- | --- | --- | --- | --- | --- | --- | --- | --- |
|  | **COVID Positive (n=183,410)** | **COVID Negative (n=204,367)** | **ARI (n=86,463)** | **SMD** | **COVID Positive** | **COVID Negative** | **ARI** | **SMD** |
|  |  |  |  |  |  |  |  |  |
| Gender | n (%) | n (%) | n (%) |  | % | % | % |  |
| Female | 90,039 (49.1) | 98,893 (48.4) | 42,113 (48.7) | 0.009 | 48.7 | 48.7 | 48.7 | 0.003 |
| Male | 93,343 (50.9) | 105,437 (51.6) | 44,336 (51.3) |  | 51.3 | 51.3 | 51.3 |  |
| Age |  |  |  |  |  |  |  |  |
| <1 | 20,773 (11.3) | 33,202 (16.2) | 16,235 (18.8) | 0.197 | 14.9 | 14.8 | 14.8 | 0.003 |
| 1 to 4 | 45,242 (24.7) | 59,268 (29.0) | 25,936 (30.0) |  | 27.5 | 27.5 | 27.6 |  |
| 5 to 9 | 42,570 (23.2) | 41,643 (20.4) | 18,385 (21.3) |  | 21.6 | 21.6 | 21.6 |  |
| 10 to 17 | 74,825 (40.8) | 70,254 (34.4) | 25,907 (30.0) |  | 36.1 | 36.1 | 35.9 |  |
| Race/Ethnicity |  |  |  |  |  |  |  |  |
| American Indian or Alaska Native Non-Hispanic | 955 (0.5) | 1,601 (0.8) | 534 (0.6) | 0.114 | 0.7 | 0.6 | 0.7 | 0.005 |
| Asian Non-Hispanic | 7,936 (4.3) | 7,542 (3.7) | 2,956 (3.4) |  | 3.9 | 3.9 | 3.9 |  |
| Black or African American Non-Hispanic | 28,753 (15.7) | 29,851 (14.6) | 12,432 (14.4) |  | 15 | 15 | 14.9 |  |
| Hispanic or Latino Any Race | 39,395 (21.5) | 52,388 (25.6) | 23,942 (27.7) |  | 24.4 | 24.5 | 24.4 |  |
| Native Hawaiian or Other Pacific Islander Non-Hispanic | 240 (0.1) | 314 (0.2) | 132 (0.2) |  | 0.1 | 0.1 | 0.1 |  |
| White Non-Hispanic | 86,823 (47.3) | 88,261 (43.2) | 37,434 (43.3) |  | 44.7 | 44.7 | 44.9 |  |
| Other Non-Hispanic | 1,683 (0.9) | 2,278 (1.1) | 846 (1.0) |  | 1 | 1 | 1 |  |
| Unknown | 17,625 (9.6) | 22,132 (10.8) | 8,187 (9.5) |  | 10.2 | 10.1 | 10.1 |  |
| BMI Category (calculated on those with age greater than 2 years) | | | | | | | | |
| 0. Age less than 2 | 35,170 (19.2) | 55,786 (27.3) | 26,155 (30.2) | 0.227 | 24.8 | 24.7 | 24.8 | 0.003 |
| 0. Missing | 57,149 (31.2) | 54,083 (26.5) | 17,202 (19.9) |  | 27 | 27 | 27 |  |
| 1. Underweight | 3,469 (1.9) | 4,601 (2.3) | 1,783 (2.1) |  | 2.1 | 2.1 | 2.1 |  |
| 2. Normal weight | 47,368 (25.8) | 50,083 (24.5) | 22,260 (25.7) |  | 25.3 | 25.3 | 25.3 |  |
| 2. Overweight | 15,212 (8.3) | 15,328 (7.5) | 7,207 (8.3) |  | 8 | 8 | 8 |  |
| 3. Class I Obesity | 15,018 (8.2) | 14,701 (7.2) | 7,185 (8.3) |  | 7.8 | 7.8 | 7.7 |  |
| 4. Class II Obesity | 6,147 (3.4) | 5,965 (2.9) | 2,939 (3.4) |  | 3.2 | 3.2 | 3.2 |  |
| 5. Class III Obesity | 3,870 (2.1) | 3,810 (1.9) | 1,729 (2.0) |  | 2 | 2 | 2 |  |
| Treatment |  |  |  |  |  |  |  |  |
| Paxlovid | 711 (0.4) | 0 (0.0) | <20 | 0.061 | 0.4 | 0 | <20 | 0.061 |
| Remdesivir | 421 (0.2) | <20 | 0 (0.0) | 0.046 | 0.2 | <20 | 0 | 0.047 |
| Corticosteroids | 9,460 (5.2) | 21,416 (10.5) | 7,826 (9.1) | 0.133 | 5.2 | 10.7 | 9 | 0.137 |
| Disease Severity | | | | | | | | |
| Hospitalization | 4,877 (2.7) | 18,210 (8.9) | 2,350 (2.7) | 0.18 | 2.9 | 8.9 | 2.8 | 0.174 |

**Table S5.** Number at risk by viral variant for COVID-19 positive, COVID-19 negative and ARI patients

|  | **COVID Positive** | **COVID Negative** | **ARI** |
| --- | --- | --- | --- |
| Ancestral Variant |  |  |  |
| 0 days | 16,926 | 96,137 | 34,351 |
| 180 days | 15,228 | 71,346 | 31,041 |
| 365 days | 14,413 | 60,665 | 28,747 |
| 548 days | 12,922 | 46,509 | 24,814 |
| 730 days | 10,179 | 31,805 | 18,608 |
| Alpha Variant |  |  |  |
| 0 days | 68,825 | 223,275 | 52,691 |
| 180 days | 60,931 | 155,798 | 44,119 |
| 365 days | 54,469 | 113,633 | 35,324 |
| 548 days | 41,706 | 71,146 | 22,624 |
| 730 days | 12,859 | 17,958 | 7,045 |
| Delta Variant |  |  |  |
| 0 days | 66,525 | 174,915 | 50,003 |
| 180 days | 52,442 | 91,198 | 34,103 |
| 365 days | 35,715 | 51,789 | 21,600 |
| 548 days | 844 | 2,424 | 1,220 |
| 730 days | *NA* | *NA* | *NA* |
| Omicron Variant |  |  |  |
| 0 days | 183,409 | 204,366 | 86,462 |
| 180 days | 97,960 | 59,882 | 26,635 |
| 365 days | 20,488 | 9,381 | 4,100 |
| 548 days | *NA* | *NA* | *NA* |
| 730 days | *NA* | *NA* | *NA* |

**Table S6.** Number at risk by viral variant and hospitalization for COVID-19 positive, COVID-19 negative and ARI patients

|  | **COVID Positive Non-Hospitalized** | **COVID Negative Non-Hospitalized** | **ARI Non-Hospitalized** | **COVID Positive Hospitalized** | **COVID Negative Hospitalized** | **ARI Hospitalized** |
| --- | --- | --- | --- | --- | --- | --- |
| Ancestral Variant |  |  |  |  |  |  |
| 0 days | 16,209 | 84,098 | 33,320 | 716 | 12,039 | 1,031 |
| 180 days | 14,587 | 61,824 | 30,137 | 641 | 9,717 | 913 |
| 365 days | 13,827 | 52,564 | 27,932 | 586 | 8,472 | 835 |
| 548 days | 12,399 | 40,130 | 24,113 | 522 | 6,932 | 735 |
| 730 days | 9,763 | 27,281 | 18,050 | 416 | 5,229 | 609 |
| Alpha Variant |  |  |  |  |  |  |
| 0 days | 67,057 | 204,475 | 51,769 | 1,768 | 18,800 | 922 |
| 180 days | 59,416 | 141,880 | 43,393 | 1,516 | 14,140 | 741 |
| 365 days | 53,090 | 103,062 | 34,764 | 1,382 | 10,981 | 613 |
| 548 days | 40,628 | 64,462 | 22,268 | 1,081 | 7,286 | 423 |
| 730 days | 12,558 | 16,207 | 6,935 | 305 | 2,480 | 183 |
| Delta Variant |  |  |  |  |  |  |
| 0 days | 64,712 | 162,414 | 48,930 | 1,812 | 12,500 | 1,072 |
| 180 days | 50,956 | 83,282 | 33,351 | 1,487 | 8,103 | 809 |
| 365 days | 34,648 | 47,022 | 21,082 | 1,067 | 5,140 | 607 |
| 548 days | 783 | 2,022 | 1,177 | 61 | 883 | 138 |
| 730 days | *NA* | *NA* | *NA* | *NA* | *NA* | *NA* |
| Omicron Variant |  |  |  |  |  |  |
| 0 days | 178,068 | 186,257 | 84,035 | 5,341 | 18,109 | 2,427 |
| 180 days | 95,336 | 53,750 | 25,905 | 2,628 | 6,353 | 771 |
| 365 days | 19,894 | 8,568 | 4,001 | 599 | 1199 | 154 |
| 548 days | *NA* | *NA* | *NA* | *NA* | *NA* | *NA* |
| 730 days | *NA* | *NA* | *NA* | *NA* | *NA* | *NA* |

**Table S7.** Weighted count of censoring events by viral variant for COVID-19 positive, COVID-19 negative and ARI patients

|  | **Reason for Censor** | **Total** | **COVID-19 Positive** | **COVID-19 Negative** | **ARI** |
| --- | --- | --- | --- | --- | --- |
| Ancestral | ARI | 15,996 | 0 | 15,996 | 0 |
|  | COVID-19 | 16,205 | 0 | 8,928 | 7,277 |
|  | Deceased | 377 | 39 | 295 | 44 |
|  | Lost to Followup | 51,481 | 6,739 | 35,986 | 8,757 |
| Alpha variant | ARI | 41,911 | 0 | 41,912 | 0 |
|  | COVID-19 | 35,631 | 0 | 23,497 | 12,135 |
|  | Deceased | 501 | 70 | 374 | 56 |
|  | Lost to Followup | 23,0063 | 56,002 | 140,199 | 33,863 |
| Delta variant | ARI | 30,585 | 0 | 30,585 | 0 |
|  | COVID-19 | 29,035 | 0 | 18,897 | 10,138 |
|  | Deceased | 255 | 49 | 186 | 20 |
|  | Lost to Followup | 218,064 | 66,332 | 112,086 | 39,647 |
| Omicron | ARI | 22,696 | 0 | 22,666 | 0 |
|  | COVID-19 | 21,515 | 0 | 119,43 | 9,572 |
|  | Deceased | 346 | 107 | 206 | 33 |
|  | Lost to Followup | 408,500 | 183,152 | 148,195 | 77,153 |

**Table S8.** Total uncensored and unweighted cases of new diabetes across all groups and proportion of cases (total cases over number at risk at T0) by variant

| **Variant Era** |  | **COVID Positive** | **COVID Negative** | **ARI** |
| --- | --- | --- | --- | --- |
| Ancestral |  | 16,926 | 96,137 | 34,351 |
|  | Cases | 70 | 310 | 75 |
|  | Proportion | 0.0041 | 0.0032 | 0.0022 |
| Alpha variant |  | 68,825 | 223,275 | 52,691 |
|  | Cases | 188 | 536 | 83 |
|  | Proportion | 0.0027 | 0.0024 | 0.0016 |
| Delta variant |  | 66,525 | 174,915 | 50,003 |
|  | Cases | 100 | 223 | 47 |
|  | Proportion | 0.0015 | 0.0013 | 0.0010 |
| Omicron |  | 183,409 | 204,366 | 86,462 |
|  | Cases | 181 | 194 | 48 |
|  | Proportion | 0.0010 | 0.0010 | 0.0006 |

**Figure S9.** Cumulative incidence of death in pediatric patients after COVID-19 compared to COVID-19 negative and ARI controls by COVID-19 variant and time period


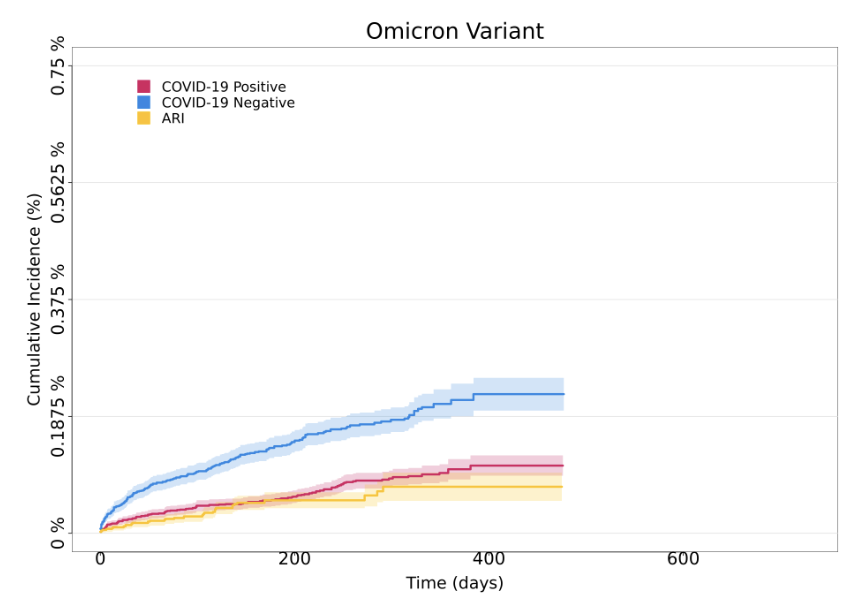

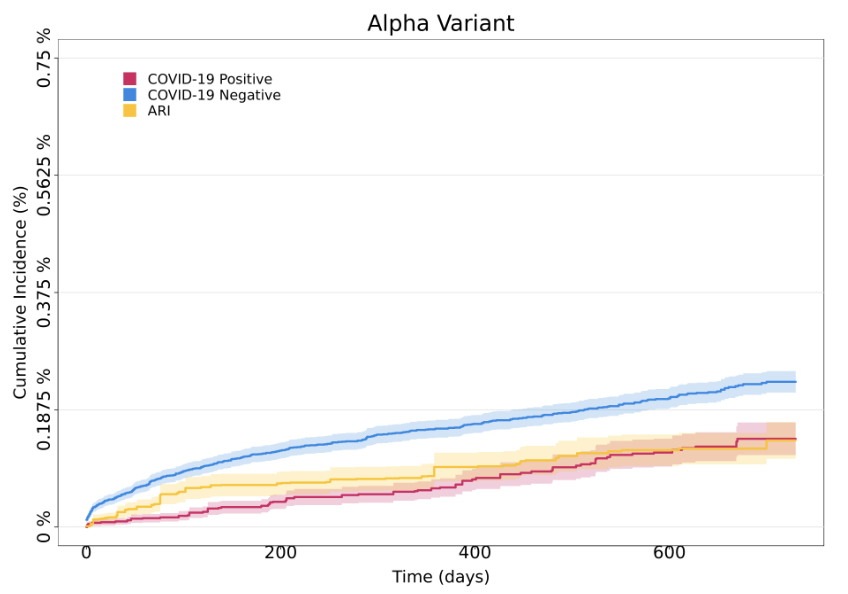

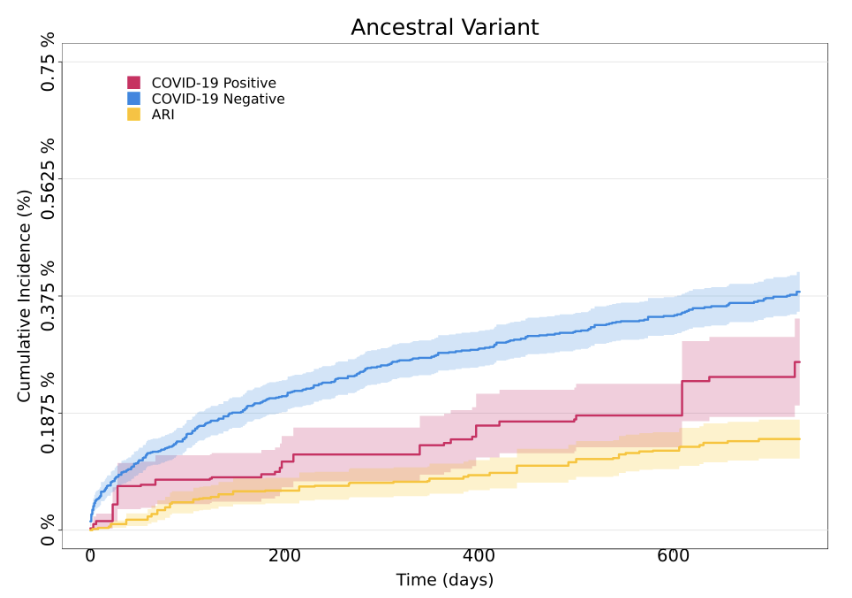

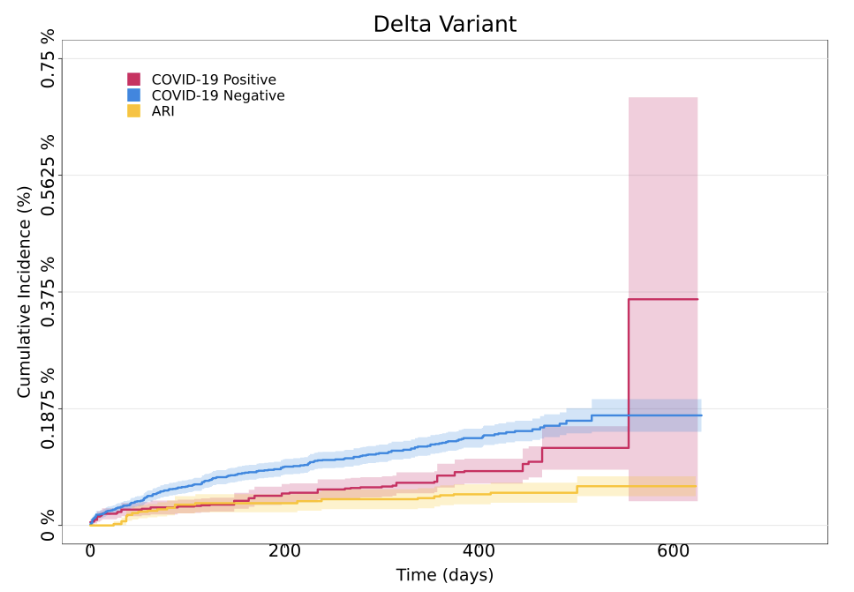


**Figure S10.** Cumulative incidence of diabetes in hospitalized and non-hospitalized

pediatric patients after COVID-19 compared to COVID-19 negative and ARI controls by COVID-19 variant and time period


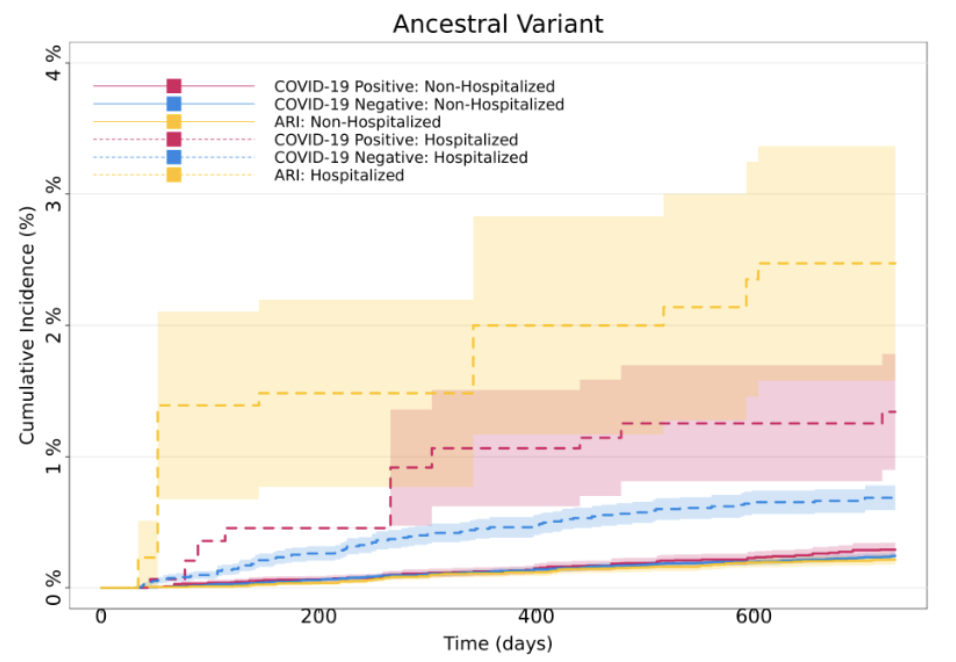

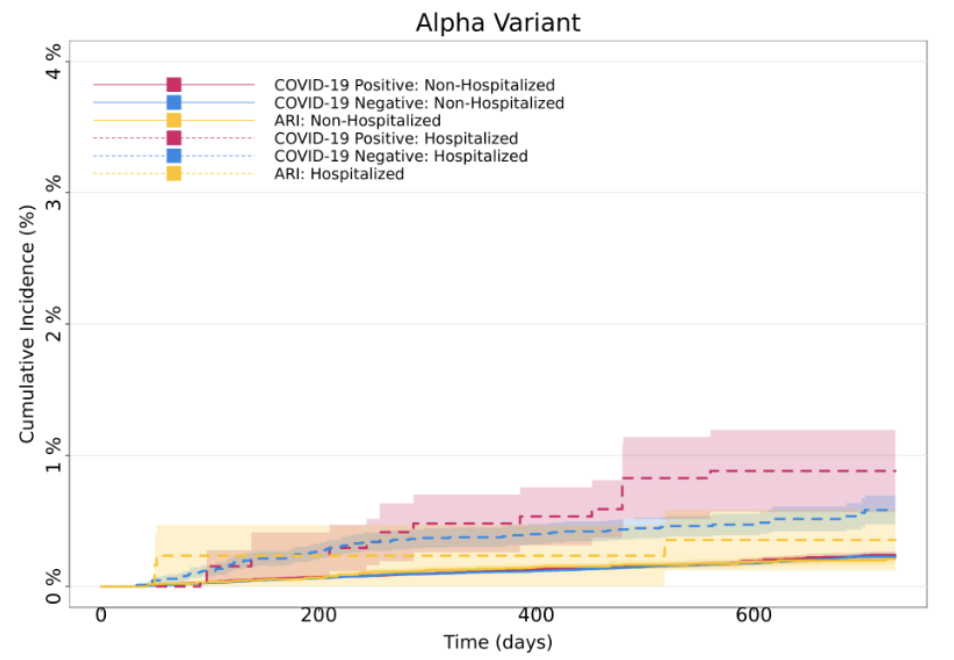

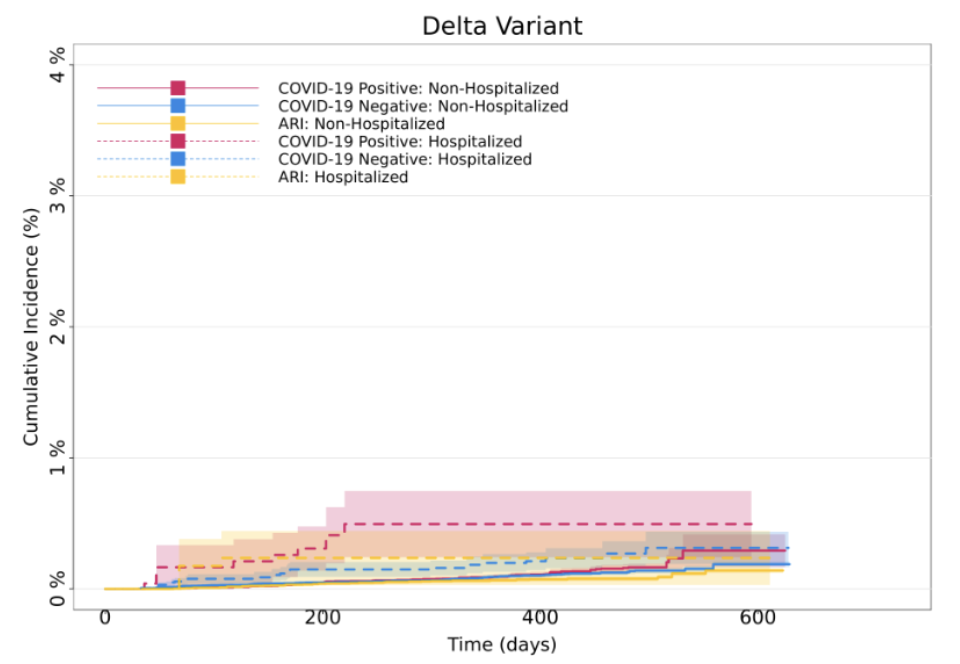

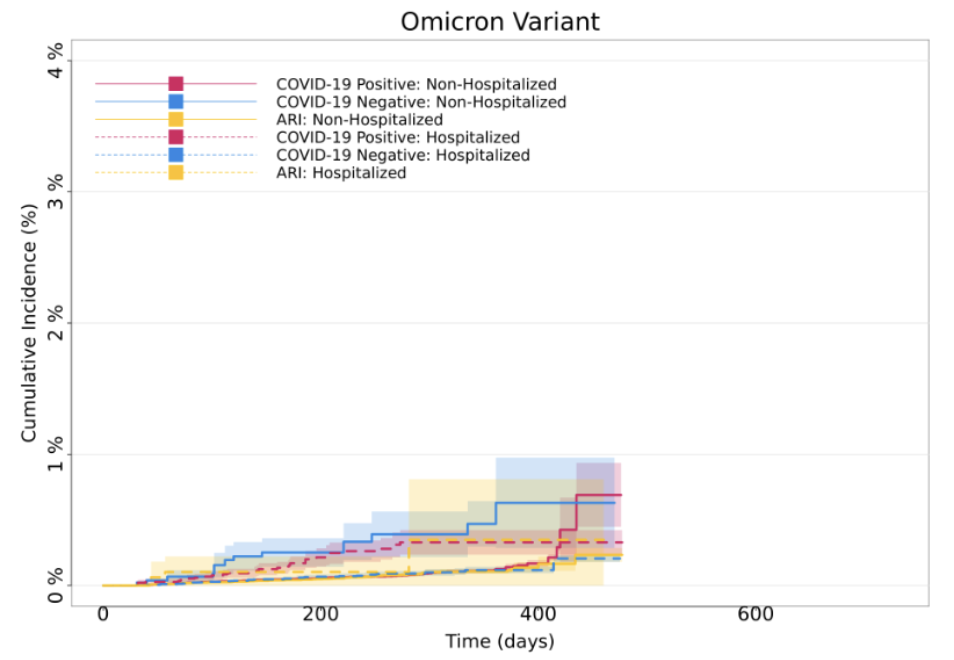


**Table S11.** Cumulative incidence of diabetes by viral variant for COVID-19 positive, COVID-19 negative and ARI patients per 10,000 patients

|  | **COVID Positive** | **COVID Negative** | **ARI** |
| --- | --- | --- | --- |
| Ancestral Variant |  |  |  |
| 180 days | 8 | 8 | 8 |
| 365 days | 17 | 17 | 16 |
| 548 days | 25 | 24 | 22 |
| 730 days | 33 | 30 | 28 |
| Alpha Variant |  |  |  |
| 180 days | 7 | 8 | 6 |
| 365 days | 13 | 14 | 14 |
| 548 days | 18 | 19 | 18 |
| 730 days | 26 | 26 | 21 |
| Delta Variant |  |  |  |
| 180 days | 4 | 6 | 4 |
| 365 days | 11 | 10 | 7 |
| 548 days | 29 | 17 | 12 |
| 730 days | *NA* | *NA* | *NA* |
| Omicron Variant |  |  |  |
| 180 days | 6 | 6 | 6 |
| 365 days | 14 | 13 | 13 |
| 548 days | *NA* | *NA* | *NA* |
| 730 days | *NA* | *NA* | *NA* |
